# Enhancement of Pneumocandin B_0_ Production in *Glarea lozoyensis* by Low-Temperature Adaptive Laboratory Evolution

**DOI:** 10.3389/fmicb.2018.02788

**Published:** 2018-11-21

**Authors:** Ping Song, Ke Zhang, Sen Zhang, Bao-Qi Huang, Xiao-Jun Ji, Lu-Jing Ren, Song Gao, Jian-Ping Wen, He Huang

**Affiliations:** ^1^Jiangsu Collaboration Innovation Center of Chinese Medical Resources Industrialization, College of Pharmacy, Nanjing University of Chinese Medicine, Nanjing, China; ^2^School of Chemical Engineering and Technology, Department of Biochemical Engineering, Tianjin University, Tianjin, China; ^3^Jiangsu Synergetic Innovation Center for Advanced Bio-Manufacture, College of Biotechnology and Pharmaceutical Engineering, Nanjing Tech University, Nanjing, China; ^4^Jiangsu Key Laboratory of Marine Bioresources and Environment, Huaihai Institute of Technology, Lianyungang, China

**Keywords:** fatty acid synthesis regulation, membrane permeability, adaptive evolution, enzyme activity, *Glarea lozoyensis*, pneumocandin B_0_

## Abstract

The production of pneumocandin B_0_ is limited by feedback inhibition. Here, low-temperature adaptive laboratory evolution (ALE) was used to improve the production capacity of *Glarea lozoyensis* by enhancing its membrane permeability. After 50 cycles of ALE, the pneumocandin B_0_ production of the endpoint strain (ALE50) reached 2131 g/L, which was 32% higher than the starting strain (ALE0). ALE50 showed a changed fatty acid composition of the cell membrane, which-+h increased its permeability by 14%, which in turn increased the secretion ratio threefold. Furthermore, ALE50 showed increased intracellular proline and acetyl-CoA concentrations, superoxide dismutase (SOD), and catalase (CAT) activity, as well as total antioxidant capacity. The slight biomass decrease in ALE50 was accompanied by decreased isocitrate dehydrogenase (ICDH) and glucose-6-phosphate dehydrogenase (G6PDH) activity. Finally, a putative model of the accumulation and secretion of pneumocandin B_0_ in ALE50 was established. ALE is a promising method to release intracellular feedback inhibition.

## Introduction

Pneumocandin B_0_ is a lipohexapeptide composed of a 10R,12S-dimethylmyristoyl side chain and a hexapeptide core, which is produced by the fungus *Glarea lozoyensis* (Li et al., 2015). Caspofungin, a semi-synthetic derivative of pneumocandin B_0_, is the first member of the echinocandins family approved of the treatment of fungal infection by the FDA. Genomic and metabolomic approaches have already been used to improve pneumocandin biosynthesis (Youssar et al., 2012; Chen et al., 2013, 2015; Song et al., 2018). Pneumocandin B_0_ is mainly biosynthesized and accumulated in the mycelia (Balkovec et al., 2014). However, it is challenging to achieve high pneumocandin B_0_ productivity inside the mycelia due to feedback inhibition.

The regulation and enhancement of membrane permeability is one of the most important methods to relieve feedback inhibition. For example, low-intensity ultrasound technology was used to improve the production of L-lactic acid and fibrinolytic enzymes by enhancing cell membrane permeability (Avhad and Rathod, 2014; Dahroud et al., 2016). The addition of the surfactants Triton X-100, CHAPS, Tween-80, and sodium taurocholate to cultures of *Clostridium thermosulfurogenes* SV2, respectively, resulted in 140%, 34%, 88%, and 28% more thermostable β-amylase and 114%, 146%, 47%, and 28% more pullulanase (Reddy et al., 1999). The addition of 2 U/mL penicillin-G after 9 h incubation resulted in the destruction of the integrity of the cell membrane and maximum glutamate titers (Nampoothiri and Pandey, 1998). Although these previous studies have proposed several regulation strategies and the production of the target metabolites has been improved, the time point of fermentation control is difficult to determine accurately, which leads to unstable production. Thus, a strain with a higher intrinsic ability to excrete metabolites would make it easier to control the pneumocandin B_0_ production process.

Adaptive laboratory evolution (ALE) has been widely utilized as a tool for developing new biological and phenotypic functions and exploring strain improvement in synthetic biology (Dragosits and Mattanovich, 2013). Specifically, ALE has been utilized to evolve strains that are better adapted to defined conditions, such as a certain carbon source, energy source, or environmental stress. ALE with light-emitting diodes was used to enhance the carotenoid biosynthesis in *Dunaliella salina* (Fu et al., 2013). ALE of *Schizochytrium* sp. under continuous high-oxygen stimulation was used to enhance docosahexaenoic acid synthesis (Sun et al., 2016). In a low-temperature environment, the membrane phase transition caused by the dehydration and peroxidation of lipids caused by excess of reactive oxygen species (ROS) will result in an increase of membrane permeability (Weinstein et al., 2000). Thus, low-temperature adaptation might induce cells to excrete more pneumocandin B_0_.

In this work, the ALE experiment was sustained for 50 cycles under continuous low-temperature conditions. The changes of the growth properties of *G. lozoyensis* during the ALE experiment were analyzed. In addition, the yield of pneumocandin B_0,_ the activity of key enzymes, and fatty acid composition, as well as membrane fluidity and permeability of the endpoint strain and the starting strain, were compared under non-ALE normal fermentation conditions. The aim of this study was to provide a new method based on ALE for the improvement of the productivity of pneumocandin B_0_ by *G. lozoyensis*, but this approach may also be useful in other fungal species that are used for the fermentation of intracellular products.

## Materials and Methods

### Microorganism and Media

*Glarea lozoyensis* CCTCC M2014416 was deposited in the China center for type culture collection (Wuhan, China) (Qin et al., 2016). This strain was used as the starting strain and also named ALE0 in this study. The media were the same as in our previous study (Song et al., 2018). The seed medium contained 40 g glucose, 20 g soybean powder, 1 g KH_2_PO_4_, 0.01 g FeSO_4_⋅7H_2_O, 0.01 g MnSO_4_⋅H_2_O, 0.002 g ZnSO_4_⋅7H_2_O, 0.001 g CaCl_2_⋅2H_2_O, 0.00056 g HBO_3_, 0.00025 g CuCl_2_⋅2H_2_O, and 0.00003 g (NH_4_)_5_MO_7_O_24_⋅4H_2_O in 1 L distilled water, and the initial pH was adjusted to 5.0. The fermentation medium contained 80 g mannitol, 20 g glucose, 20 g peptone, and 2.5 g K_2_HPO_4_ in 1 L distilled water, and the initial pH was adjusted to 6.8.

### ALE Experiment

Adaptive laboratory evolution was based on a long-term serial transfer procedure using low temperature as a stress inducer in the seed medium. The ALE process were as follows: (1) 5 mL of the seed culture of mycelia were added to 5 mL cryopreservation solution (16% glycerin + 40%PEG-6000) and stored at -80°C for 5 days to obtain the frozen mycelia; (2) 10 mL of the above frozen mycelia were added to 50 mL of seed medium and cultured at 15°C, 220 rpm for 10 days in 250-mL shake flasks; (3) the shake flasks were shifted to 25°C, 220 rpm for another 5 days of culture, and the ALE strain in this cycle was obtained; (4) the samples with the highest DCW among three parallel cultures were added to the frozen storage solution to repeat step (1). ALE was conducted repeatedly in a 20-day cycle. Every 10 cycles, ALE strains were named ALE10, ALE20, ALE30, and ALE40, respectively. The ALE 50 strain was the endpoint strain.

### Fermentation Condition

To investigate the pneumocandin B_0_ production capacity of the ALE strains, fermentations of the strains from ALE0 to ALE50 were conducted at the same batch as follows: 10 mL of the frozen mycelia of ALE strains were added to 250-mL shake flasks with 50 mL of seed medium and cultured at 25°C and 220 rpm for 168 h to obtain seed cultures. Fermentation was carried out in 250-mL flasks containing 50 mL of fermentation medium inoculated with 10% (v/v) of the above seed cultures and incubated at 25°C and 220 rpm for 432 h.

### Determination of the Fatty Acid Composition of the Cell Membrane and of the Intercellular Compartment

For extracting the fatty acids of the cell membrane, the mycelia in the fermentation broth were harvested by centrifugation at 8000 g for 10 min. The fatty acids in the cell membrane of the mycelia were extracted, purified, and methylated according to the method described by Wang et al. (2013). The resulting sample dissolved in *n*-hexane was collected for GC–MS analysis.

For the extraction of the fatty acids of the intracellular compartment, the method was described in our previous study (Ren et al., 2009). To determine the fatty acids composition, GC-MS analysis was used as described by our previous study (Song et al., 2018).

### Analysis of the Biomass, Glucose, Mannitol, Acetyl-CoA, and Proline Concentrations

The analysis of the biomass, glucose, and mannitol concentrations was performed using the methods described in our previous study (Song et al., 2018). The analysis of intracellular acetyl-CoA levels was performed using the method described by Xia et al. (2013). The analysis of intracellular proline levels was performed using the HPLC method described in our previous study (Song et al., 2013).

### Analysis of the Pneumocandin B_0_ Concentration

The extraction of total pneumocandin B_0_ (intracellular and extracellular) was conducted using the method described in our previous study (Song et al., 2018). An aliquot comprising 1 mL of the cell-containing fermentation broth was extracted with 4 mL ethyl alcohol at room temperature on a Vortex Genie mixer (Scientific Industries, United States) for 10 min. The extract was then centrifuged at room temperature and 8000 g for 5 min, and the supernatant was analyzed by HPLC. To determine the extracellular pneumocandin B_0_ concentration, the cell-containing fermentation broth centrifuged at room temperature and 8000 g for 5 min and the supernatant was analyzed by HPLC, too. The intracellular pneumocandin B_0_ concentration was calculated as the total minus the extracellular concentration.

### Extraction and Assay of Enzyme Activity

Mycelia were collected by centrifugation at 15,000 *g* for 10 min at 4°C and washed with distilled water, followed by disruption *via* ultrasonication in pre-cooled PBS buffer containing 0.5 mM phenylmethylsulfonyl fluoride (Sigma, United States) at 4°C for 8 min. The supernatant obtained by centrifugation at 4°C and 15,000 g for 10 min was used to determine enzyme activities.

The activities of ATP:citrate lyase (ACL), isocitrate dehydrogenase (ICDH), malic enzyme (ME), and glucose-6-phosphate dehydrogenase (G6PDH) were determined as described in our previous study (Song et al., 2018). The activities of the antioxidant enzymes catalase (CAT) and total superoxide dismutase (SOD) were measured using commercially available kits [Nanjing Jiancheng Bioengineering Institute (NJBI), China]. One unit of CAT activity was defined as the amount of enzyme that decomposes 1.0 μmol of H_2_O_2_ per minute under the assay described in the manufacturer’s manual conditions. SOD activity was evaluated by measuring the inhibition of the oxidation of cytochrome C by xanthine/xanthine oxidase at 550 nm. One arbitrary unit of SOD activity was defined as the amount of enzyme that produced 50% inhibition. Protein concentrations were determined using the Bradford assay with bovine serum albumin as the standard (Bradford, 1976).

### Analysis of Malondialdehyde (MDA), Total Antioxidant Capacity (T-AOC), and Reactive Oxygen Species (ROS)

Intracellular MDA and T-AOC levels were determined using commercially available assay kits (NJBI, China) according to the manufacturer’s instructions. The intracellular ROS levels were determined spectrophotometrically using 2′,7′-dichlorofluorescein diacetate (DCFH-DA; Sigma, United States) as previously described by Kenne et al. (2018).

### Determination of Membrane Permeabilization

Membrane permeabilization was analyzed using the 1-*N*-phenylnaphthylamine (NPN) as described by Gong et al. (2017) with some modification. The culture broth was centrifuged at 8000 g and 4°C for 5 min, the collected wet mycelia were washed two times and re-suspended in PBS buffer, and the resulting suspension diluted 100 times. Then, 2.5 mL of the diluted cell suspension was mixed with 20 μL of 1 mM NPN (Sigma, United States), and the fluorescence was recorded immediately as a function of time due to partitioning of NPN into the membrane. The fluorescence was recorded using a SpectraMax M3 fluorescence spectrophotometer (Molecular Devices, United States) with an excitation wavelength of 350 nm and an emission wavelength of 420 nm.

### Determination of Membrane Fluidity

Membrane fluidity was determined continuously by measuring fluorescence anisotropy in intact whole cells using the hydrophobic probe 1,6-diphenyl-1,3,5-hexatriene (DPH). A total of 5 μL 1.5 mM DPH (Sigma, United States) solution was added to 2.5 mL of cell suspension to achieve a final probe concentration of 3.0 μM. The fluorescence was recorded using a SpectraMax M3 fluorescence spectrophotometer with an excitation wavelength of 350 nm and an emission wavelength of 400 nm. Anisotropy values (*r*) were automatically calculated by the spectrofluorometer as described by Capozzi et al. (2011).

## Results and Discussion

### Growth Properties and Cell Membrane Characteristics of *G. lozoyensis* During ALE

**FIGURE 1:**
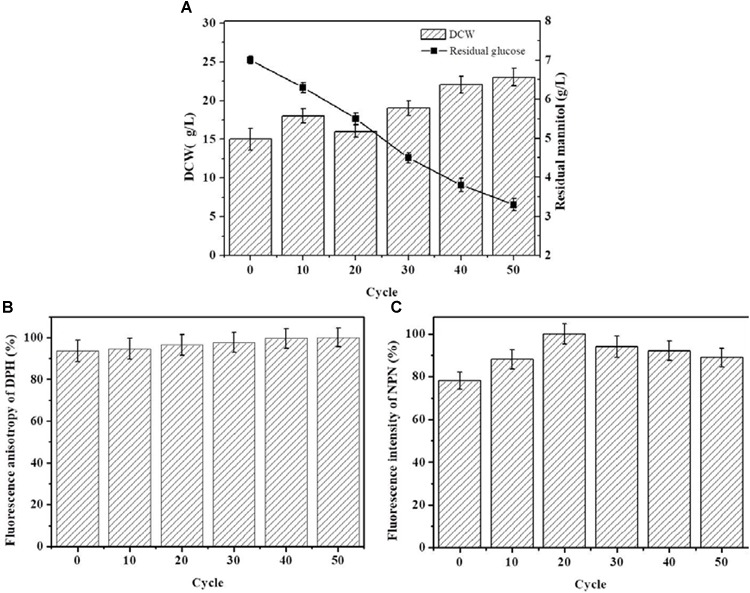
The growth properties and cell membrane characteristics of *G. lozoyensis* during the ALE experiment. **(A)** The growth property es of *G. lozoyensis* during ALE experiments. **(B)** Changes in membrane fluidity during the ALE experiments. The fluorescence anisotropy of DPH is inversely proportional to membrane fluidity. **(C)** Changes in membrane permeability during the ALE experiments. The fluorescence intensity of NPN is directly proportional to membrane permeability. Each experiment was performed three times from independent cultures, and error bars represent standard deviation.

The starting strain ALE0 of *G. lozoyensis* was cultivated under low temperature conditions for 50 cycles, and ALE strains were collected at the end of each 10 cycles to monitor the glucose consumption and dry cell weight (DCW) during ALE (Figure 1A), which was recognized as first indicators of the success or failure of ALE. With the extension of the ALE time and the natural accumulation of adaptive mutations, the strains’ consumption of glucose increased 1.2-fold. The DCW of the endpoint strain ALE50 reached 23 g/L, which was 53% higher than that of the starting strain ALE0. However, there was hardly any pneumocandin B_0_ production in the ALE process, with a yield below 50 mg/L (data not shown).

Figure 1B shows the change in membrane fluidity of the strains during ALE. During the entire ALE process, the membrane fluidity in ALE strains did not show obvious changes. Figure 1C shows the changes of membrane permeability of the strains in the course of adaptive evolution. The membrane permeability first increased and then decreased in the process of ALE. The permeability of the endpoint strain ALE50 was 12% higher than the starting strain ALE0.

The fatty acid composition of the cell membrane during ALE is shown in Table 1. The major fatty acids produced were myristic acid (C14:0), palmitic acid (C16:0), stearic acid (C18:0), eicosanoic acid (20:0), hexadecenoic acid (16:1), oleic acid (C18:1), linoleic acid (C18:2 and C18:3n3), and dihomo-γ-linolenic (C20:3n6). The fatty acid composition and total fatty acid content did not vary. However, the ratio of unsaturated/saturated fatty acid (UFA/SFA) and the index of unsaturated fatty acids (IUFA) increased significantly after 20 cycles of adaptive evolution. The ratio of UFA/SFA and IUFA of ALE50 reached 3.75 and 153.93, respectively, representing increases of 68 and 18% compared with ALE0. This indicates that the adaptation to low temperature has changed the UFA/SFA ratio among the membrane lipids and further affected the fluidity and permeability of the cell membrane.

**Table 1:** The membrane composition of *G. lozoyensis* during ALE.

Fatty acid composition	ALE strains					
	
	ALE0	ALE10	ALE20	ALE30	ALE40	ALE50
Saturated fatty acids						
Myristic acid (C14:0)	3.51 ± 0.18	3.50 ± 0.18	3.33 ± 0.17	2.89 ± 0.14	2.88 ± 0.14	2.46 ± 0.12
Palmitic acid (C16:0)	14.52 ± 0.73	14.53 ± 0.73	14.49 ± 0.72	13.79 ± 0.69	12.62 ± 0.63	11.45 ± 0.57
Stearic acid (C18:0)	10.60 ± 0.53	10.63 ± 0.53	9.69 ± 0.48	9.13 ± 0.46	6.06 ± 0.30	6.22 ± 0.31
Eicosanoic acid (C22:0)	2.36 ± 0.12	2.33 ± 0.12	1.97 ± 0.10	2.90 ± 0.15	1.95 ± 0.10	1.74 ± 0.09
Unsaturated fatty acids						
Hexadecenoic acid (C16:1)	11.90 ± 0.60	12.00 ± 0.60	12.26 ± 0.61	12.27 ± 0.61	12.34 ± 0.62	12.32 ± 0.62
Oleic acid (C18:1)	16.62 ± 0.83	16.71 ± 0.84	17.80 ± 0.89	17.25 ± .86	16.93 ± 0.85	17.01 ± 0.85
Linoleic acid (C18:2)	19.80 ± 0.99	19.44 ± 0.97	17.92 ± 0.90	18.56 ± 0.93	20.20 ± 1.01	21.79 ± 1.09
Linoleic acid (C18:3n3)	4.26 ± 0.21	4.75 ± 0.24	5.44 ± 0.27	5.61 ± 0.28	5.79 ± 0.29	6.19 ± 0.31
Dihomo-γ-linolenic (C20:3n6)	16.43 ± 0.82	16.11 ± 0.81	17.1 ± 0.86	17.6 ± 0.88	21.23 ± 1.06	20.82 ± 1.04
Unsaturated/saturated fatty acid ratio^a^	2.23 ± 0.11	2.23 ± 0.11	2.39 ± 0.12	2.48 ± 0.12	3.25 ± 0.16	3.75 ± 0.19
IUFA (index of unsaturated fatty acids)^b^	130.19 ± 6.51	130.17 ± 6.51	133.52 ± 6.68	136.27 ± 6.81	150.73 ± 7.54	153.94 ± 7.70


*^*a*^(C16:1 + C18:1 + C18:2 + C18:3n3 + C20:3n6)/(C14:0 + C16:0 + C18:0 + C22:0). ^*b*^The index of unsaturated fatty acids (IUFA) in the polar lipids was calculated as IUFA = (C16:1 mol % + C18:1t mol % + 2 C18:2 mol % + 3 C18:3n3 mol % + 3 C20:3n6 mol %) × 100. Each experiment was performed three times from independent cultures, and error bars represent standard deviation.*

### Cellular Antioxidant Capacity During ALE

**FIGURE 2:**
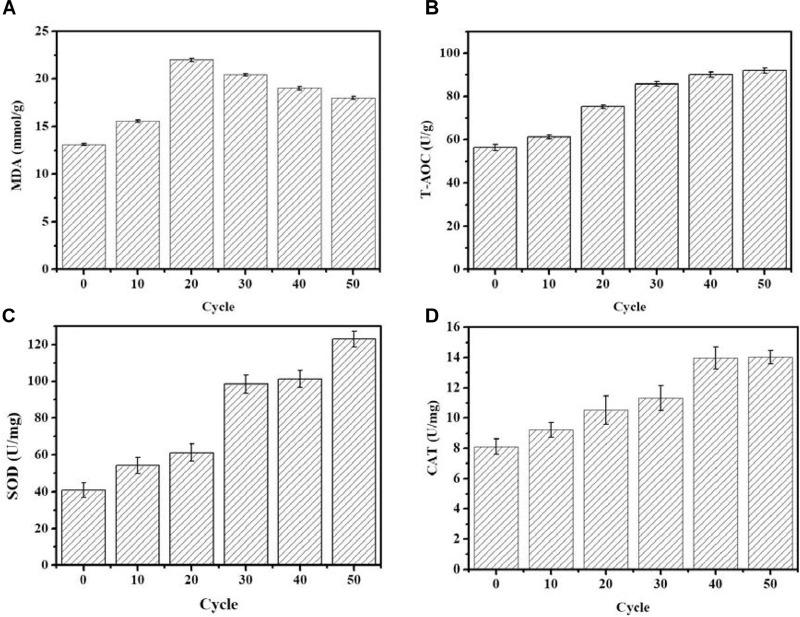
Changes of intracellular antioxidant capacity during the ALE experiment. **(A)** The concentration of MDA. **(B)** T-AOC. **(C)** The SOD enzyme activity. **(D)** The CAT enzyme activity. Each experiment was performed three times from independent cultures, and error bars represent standard deviation.

As shown in Figure 2, the MDA concentration reached the maximum value of 21.98 nmol/g in the 20th round of ALE, and subsequently gradually decreased as the ALE continued. During the ALE process, the T-AOC of the culture system increased with consecutive ALE cycles. The activities of antioxidant enzymes SOD and CAT also increased with the increase of ALE cycle numbers.

Low temperatures can increase the permeability of the cell membrane. In below-freezing environments, ice crystals first appear in the extracellular solution, which results in a difference of chemical potential between the inside and outside of the cell (Mazur, 1972). The chemical potential difference causes the water in the cell to exude through the cell membrane to maintain the balance between internal and external chemical potentials, which in turn led to cell dehydration (Mazur, 1972). Cell dehydration increases the concentration of the intracellular solution, resulting in protein damage and destruction of the cellular membrane structure, resulting in increased membrane permeability (Mazur, 1972). In addition, low temperatures can also increase cell membrane permeability through ROS. Low-temperature stress can lead to excessive accumulation of ROS, induce peroxidation of cell membrane components, destroy the cell membrane structure, and increase membrane permeability (Maksimov et al., 2017). Malondialdehyde (MDA) is the product of membrane lipid peroxidation, and its content is positively correlated with the degree of membrane lipid peroxidation (Baust et al., 2001). Antioxidant enzyme systems are the main mechanism by which organisms eliminate ROS. In the process of resisting oxidative stress, SOD and CAT usually cooperate to eliminate ROS from cells (Fridovich, 1981). T-AOC reflects the ability of intracellular antioxidants to scavenge ROS. In the face of low-temperature stress, the enzyme activity of SOD and CAT, as well as the T-AOC of *G. lozoyensis* cells increased (Figures 2B–D) to resist the increase of ROS and reduce the resulting damage to the cell membrane. ALE requires different periods of time to be effective. *Schizochytrium* sp. requires 40 adaptation cycles to cope with H_2_O_2_ stress (Sun et al., 2016). *D. salina* requires 16 cycles to adapt to light stimulation (Fu et al., 2013). A low-temperature environment affects the growth characteristics and cell membrane composition of *G. lozoyensis*, which led to the development of a specific phenotype through the 50 cycles of ALE (Figure 1A).

Low temperature did not cause obvious changes in membrane fluidity in this study (Figure 1B). Many factors can affect the membrane fluidity, including the content of unsaturated fatty acids, methoxy fatty acid, other physical properties of cell membrane and lipid composition (Denich et al., 2003; Los and Murata, 2004). It has been reported that membrane fluidity can self-regulate itself (Kitajima and Thompson, 1977). Therefore, in this study, it may be due to the self-regulation mechanism of cell membrane during ALE experiment, which makes the composition of unsaturated fatty acids in cell membrane change greatly (Table 1), but the fluidity of cell membrane did not change significantly (Figure 1B).

### Correlation Between Pneumocandin B_0_ Production and the Cell Membrane Characteristics of ALE Strains During Fermentation

**FIGURE 3:**
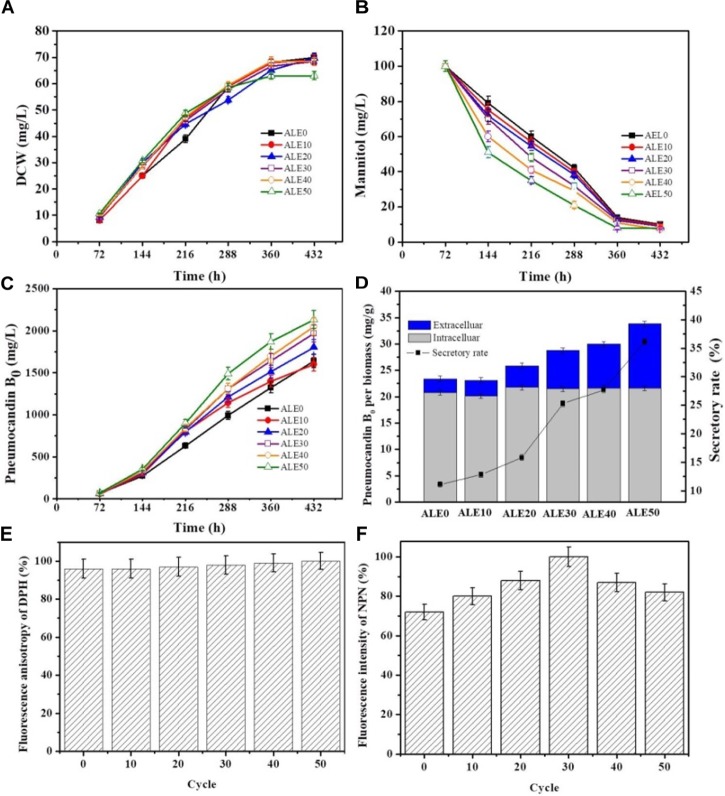
The fermentation characteristics and membrane characteristics of different ALE strains during fermentation. **(A)** DCW. **(B)** Mannitol consumption. **(C)** Total pneumocandin B_0_ concentration (including extracellular and intracellular). **(D)** The secretory rate of pneumocandin B_0_ at 432 h_._ The pneumocandin B_0_ per biomass was calculated as the pneumocandin B_0_ concentration (mg/L)/DCW (g/L). The secretory rate of pneumocandin B_0_ was calculated as extracellular/total. **(E)** Changes in membrane fluidity. The fluorescence anisotropy of DPH is inversely proportional to membrane fluidity. **(F)** Changes in membrane permeability. The fluorescence intensity of NPN is directly proportional to membrane permeability. Each experiment was performed three times from independent cultures, and error bars represent standard deviation.

To investigate the pneumocandin B_0_ production capacity of the ALE strains, the starting strain ALE0 and five ALE strains (ALE10, ALE20, ALE30, ALE40, and the endpoint strain ALE50) were cultured under fermentation condition at 25°C. The time profiles of DCW and mannitol consumption by different ALE strains are shown in Figures 3A,B. ALE50 produced only 63 g/L DCW, which was 10% less than ALE30, and 32% less than ALE0. However, compared with the starting strain ALE0, the ALE strains had significantly enhanced mannitol consumption rates during 72 h–144 h. As shown in Figure 3C, the total pneumocandin B_0_ concentration (intracellular + extracellular) of ALE50 reached 2131 g/L, representing a 30% increase over ALE0. As shown in Figure 3D, ALE0 and ALE50 had pneumocandin B_0_ secretion rates of 11% and 36%, respectively. Thus, the secretion rate of pneumocandin B_0_ in ALE50 increased threefold compared with ALE0.

The changes of the membrane phenotypes of fluidity, permeability, and fatty acid composition that appeared in the ALE process were also apparent in the fermentations at 25°C. As shown in Figures 3E,F, the membrane permeability during the fermentation of ALE50 was 14% higher than that of ALE0, while the membrane fluidity was almost unchanged. As shown in Table 2, the ratio of UFA/SFA also increased during the fermentation, similar to the observations made during ALE. Because the fermentation temperature of 25°C was higher than the ALE temperature, which was set to either -80°C or 15°C, the UFA/SFA ratio in the fermentation process was lower than that in the ALE. The cell membrane permeability of the ALE strains was consistent with the increase of extracellular pneumocandin B_0_ (Figure 3D). The increase of cell permeability promoted the excretion of pneumocandin B_0_ and relieved the intracellular product inhibition.

**Table 2:** The membrane composition of different ALE strains during fermentation.

Fatty acid composition	ALE strain	
	
(mol %)	ALE0	ALE50
Saturated fatty acids		
Myristic acid (C14:0)	4.26 ± 0.23	4.81 ± 0.24
Palmitic acid (C16:0)	17.58 ± 0.85	12.1 ± 0.65
Stearic acid (C18:0)	12.85 ± 0.60	6.18 ± 0.30
Eicosanoic acid (C22:0)	2.02 ± 0.12	1.85 ± 0.09
Unsaturated fatty acids	11.99 ± 0.55	13.14 ± 0.65
Hexadecenoic acid (C16:1)	17.74 ± 0.80	21.32 ± 1.02
Oleic acid (C18:1)	14.45 ± 0.70	18.13 ± 0.95
Linoleic acid (C18:2)	2.89 ± 0.13	4.92 ± 0.22
Linoleic acid (C18:3n3)	16.22 ± 0.85	17.55 ± 0.85
Dihomo-γ-linolenic (C20:3n6)	4.26 ± 0.25	4.81 ± 0.24
Unsaturated/saturated fatty acid ratio^a^	1.72 ± 0.05	3.01 ± 0.15
IUFA (index of unsaturated fatty acid)^b^	115.96 ± 5.55	138.13 ± 7.56


*^*a*^(C16:1 + C18:1 + C18:2 + C18:3n3 + C20:3n6)/(C14:0 + C16:0 + C18:0 + C22:0). ^*b*^The index of unsaturated fatty acids (IUFA) in the polar lipids was calculated as IUFA = (C16:1 mol % + C18:1t mol % + 2 C18:2 mol % + 3 C18:3n3 mol % + 3 C20:3n6 mol %) × 100. Each experiment was performed three times from independent cultures, and error bars represent standard deviation.*

### Activities of Key Enzymes and Concentrations of Key Metabolites in ALE0 and ALE50 During Fermentation

**FIGURE 4:**
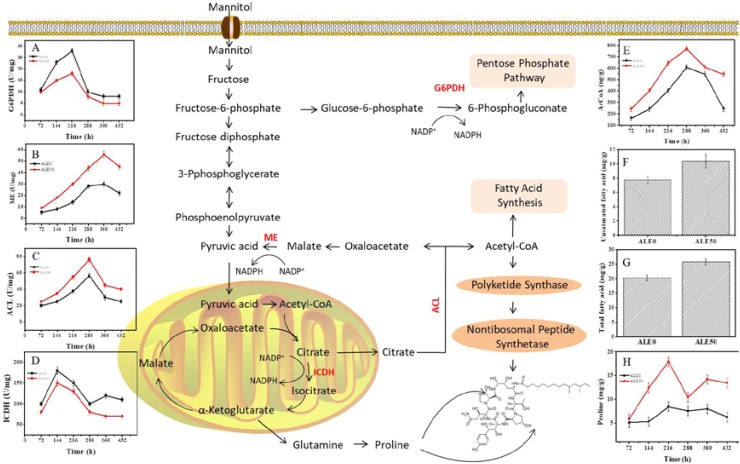
Changes of key enzyme activities of ALE strains during fermentation. **(A)** G6PDH enzyme activity. **(B)** ME enzyme activity. **(C)** ACL enzyme activity. **(D)** ICDH enzyme activity. **(E)** Acetyl-CoA concentration. **(F)** Intracellular total fatty acids at 432 h. **(G)** Intracellular unsaturated fatty acids at 432 h **(H)** Intracellular proline. Each experiment was performed three times from independent cultures and error bars represent standard deviation. Each experiment was performed three times from independent cultures, and error bars represent standard deviation.

In order to investigate whether the decrease of DCW and increase of pneumocandin B_0_ production in ALE0 and ALE50 during fermentation were related to shifts in the related metabolic pathways, the activities of key enzymes and concentrations of key metabolites in the central carbon metabolism were measured (Figure 4).

NADPH and acetyl-CoA play important roles in the synthesis of pneumocandin B_0_. G6PDH and ME activities were used to reflect the NADPH supply, given that the supply of reducing power in the form of NADPH is known to play a significant role in PKS-mediated biosynthesis and product accumulation (Song et al., 2018). The activity of G6PDH in both ALE0 and ALE50 increased with the culture time and reached its maximum specific activity of 28 and 18 U/mg total protein at 216 h (Figure 4A). The activity of ME in ALE0 and ALE50 also increased with the culture time, and reached its maximum of 30 and 56 U/mg total protein, respectively, at 360 h (Figure 4B). These results were consistent with our previous findings that G6PDH provides the NADPH in the early fermentation stage, but ME provide NADPH in the later fermentation stages (Song et al., 2018). The higher ME activity in ALE50 probably solved the problem of insufficient NADPH supply at the late stage of pneumocandin B_0_ fermentation (Song et al., 2018). ATP:citrate lyase and ICDH activities were used to reflect the acetyl-CoA supply.

Higher levels of ICDH activity and lower levels of ACL activity were observed in ALE50 (Figures 4C,D). The ACL activity of ALE0 and ALE50 reached the maximum of 56.5 and 76.5 U/mg at 288 h. Compared with ALE0, the ACL activity of ALE50 was increased by 35%. The ICDH activity of ALE0 and ALE50 reached the maximum of 180.2 and 150.4 U/mg at 144 h. Compared with the starting strain ALE0, the ICDH activity of ALE50 decreased by 17%. The higher ACL activity in ALE50 is consistent with the higher intracellular acetyl-CoA concentration in this strain (Figure 4E). At the same time, the lower ICDH enzyme activity in ALE50 means that more acetyl-CoA is available to enter the citrate pyruvate pathway at the citric acid node. Thus, more acetyl-CoA was shifted toward the synthesis of pneumocandin B_0_.

The contents of total fatty acids in ALE0 and ALE50 were 20.36 and 25.92 mg/g DCW, respectively, corresponding to a 24% increase in ALE50 (Figure 4F). The contents of UFAs in ALE0 and ALE50 were 7.72 and 10.38 mg/g DCW representing a remarkable 99% increase in ALE50 (Figure 4G). In ALE0 and ALE50, the ratios of total pneumocandin B_0_ (g/DCW)/the intracellular fatty acid (g/DCW) were 1.15 and 1.31. Therefore, in ALE0, the intracellular acetyl-CoA tends to flow toward the fatty acid synthesis pathway, whilst in ALE50, there are more intracellular pneumocandin B_0_ excreted into the broth because of the increased membrane permeability. This, in turn, promoted the flow of acetyl-CoA toward the pneumocandin B_0_ pathway instead of the fatty acid pathway. Similar phenomena were observed in the extractive fermentation of *Monascus purpureus* by adding Triton X-100, in which the increased permeability promoted the excretion of intracellular pigments resulting in a higher yield of both the total and extracellular pigments, in conjunction with a lower lipid content compared to the non-extractive batch fermentation (Gong et al., 2017).

In ALE50, intracellular proline increased by 20% (Figure 4H). Under low-temperature stress, microbes accumulated small molecules such as proline and glutathione, whereby proline acted as a source of nitrogen, carbon, and NADPH to resume growth after cold stress (Britikov et al., 1970). In addition, there are two proline residues in the peptide ring of pneumocandin B_0_, and the addition of proline can promote its synthesis (Petersen et al., 2001). Therefore, the proline produced under low-temperature stress may have promoted the formation of pneumocandin B_0_ to a certain extent.

In addition to achieving the target phenotype at the end of evolution, ALE is usually accompanied by trade-offs. For example, ALE in *Escherichia coli* increased ethanol tolerance at the expense of reduced acid resistance (Goodarzi et al., 2010). Yeast cells evolved to utilize galactose efficiently grew poorly on glucose-based media (Hong et al., 2011). Similarly, in *G. lozoyensis*, long-term culture under low-temperature stress resulted in ALE50 demonstrated slower biomass accumulation than ALE0 during fermentation (Figure 3A). The lower ICDH and G6PDH enzyme activities found in ALE50 than in ALE0 (Figures 4A,D) may explain these trade-offs. The flux flow toward the PP pathway, which provides precursors for biomass generation, was reduced, and only a small proportion of citric acid molecules entered the TCA cycle and therefore did not provide enough ATP to satisfy the energy needs of cell growth.

**FIGURE 5:**
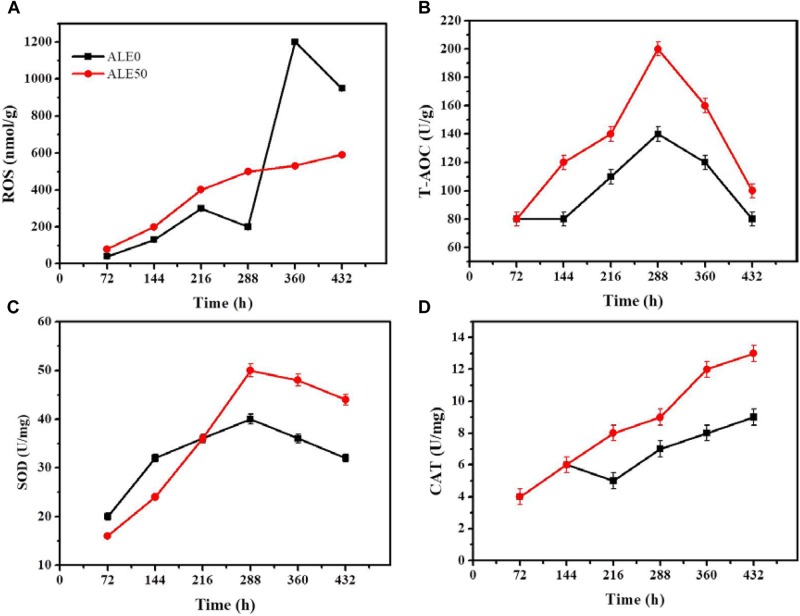
Changes antioxidant enzymes of ALE strains during fermentation. **(A)** ROS. **(B)** T-AOC. **(C)** SOD enzyme activity. **(D)** CAT enzyme activity. Each experiment was performed three times from independent cultures, and error bars represent standard deviation.

The oxidative stress exhibited by ALE50 during fermentation was similar to what was observed in the ALE process. As shown in Figure 5A, ROS peaked twice in ALE0, once at 216 h, and again at 316 h, with a maximum of 1200 nmol/g. In ALE50, the ROS reached the maximum value of 600 nmol/g at 288 h. ALE0 demonstrated higher ROS levels at 316 h, probably due to the mycelia aging (Shi et al., 2017). The total T-AOC of ALE50 was higher than that of ALE0, and increased 43% at 288 h (Figure 5B). ALE50 showed higher SOD and CAT enzyme activities, especially in the late fermentation stage, with 25% and 29% increases at 288 h (Figures 5C,D). Li et al. (2011) found that the ROS levels at 10 and 20°C were higher than at higher temperatures, and at low temperatures, the lipid content per microalgal biomass increased. These finding were different from what was found here, where low-temperature ALE strains had a lower ROS (Figure 5A). We speculate that the ALE process led to genetic changes so that the ALE50 strain adapted to the low temperature. When ALE50 was returned to the normal fermentation temperature, the adaptations related to temperature resistance, such as the higher proline concentration (Figure 4H), T-AOC, SOD, and CAT enzyme activity (Figures 5B–D) led to a better performance with lower ROS levels.

Reactive oxygen species levels and oxidative stress are essential physiological phenomena in filamentous fungi and other microorganisms (Macarisin et al., 2010; Nanou et al., 2011). On the one hand, moderate oxidative stress in the cell is an essential prerequisite for the growth and physiological metabolism of aerobic microbes, including the biosynthesis of the cell wall, proliferation, differentiation, and the formation of secondary metabolites (Reverberi et al., 2010; Hu et al., 2011; Reverberi et al., 2012; Hu et al., 2013). Similarly, the increase of ROS levels in *Aspergillus* by adding cumene hydrogen peroxide (the peak of LOOH reached 140 μg/25 mL) promoted the formation of aflatoxin B_1_ (Reverberi et al., 2006). On the other hand, excessive oxidative stress and ROS can attack biological molecules such as proteins, nucleic acids, and lipids, and thus cause oxidative damage, premature cell aging, and even death (Apel and Hirt, 2004). Cell culture experiments showed that ROS at the nanomolar concentrations could promote cell proliferation, while micromolar concentrations led to apoptosis, and millimolar levels could cause excessive oxidative damage leading directly to cell death (Panday et al., 2015). Similarly, low-temperature adaptation improves the ability of *G. lozoyensis* to maintain the levels of antioxidants and antioxidant enzymes, and thus maintain ROS levels at nanomolar concentrations, which can guarantee the synthesis of pneumocandin B_0_, avoiding excessive accumulation of ROS and the resulting oxidative damage.

**FIGURE 6:**
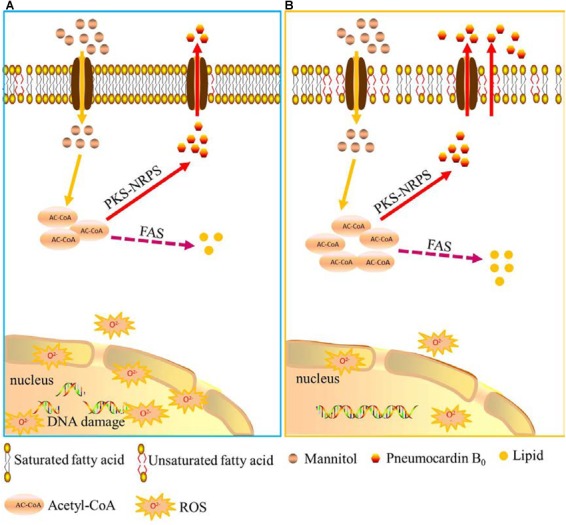
Schematic representation of the hypothesis of how low-temperature ALE improves pneumocandin B_0_ production. **(A)** A putative model of pneumocandin B_0_ accumulation and secretion in the starting strain ALE0. **(B)** A putative model of pneumocandin B_0_ accumulation and secretion in the endpoint strain ALE50.

Based on the above results and analysis, a hypothesis on how low-temperature ALE improves the yield of pneumocandin B_0_ is proposed. As shown in Figure 6, low temperature, first, increases membrane permeability, speeds up the excretion of pneumocandin B_0_, and relieves intracellular product inhibition. Second, due to the release of the inhibition effect, acetyl-CoA flux was rerouted toward the pneumocandin B_0_ synthesis pathway, and the synthesis rate of intracellular pneumocandin B_0_ was further improved. Third, low temperature promoted the accumulation of proline and increased the supply of amino acids. Finally, low-temperature stimulation improved the ability of cells to resist ROS and maintained them at nanomolar levels, which guaranteed the pneumocandin B_0_ synthesis and avoided excessive accumulation of ROS to damage the cells.

## Conclusion

The adapted strain after 50 rounds of continuous low-temperature ALE produced more pneumocandin B_0_ and lower DCW compared with the starting strain. These major performance changes were accompanied by enhanced membrane permeability, proline accumulation, ACL activity, SOD activity, and CAT activity, and reduced ICDH and G6PDH activities. In addition, the intracellular product feedback inhibition was relieved through the enhanced excretion ability, and ALE facilitated the metabolic shift from lipid accumulation to pneumocandin B_0_ yield. Therefore, ALE may be an effective strategy to further develop *G. lozoyensis* strains for industrial applications.

## Author Contributions

PS and SZ carried out the experiments and wrote the manuscript. KZ provided technical support of fermentation. B-QH provided detection of pneumocandin B_0_. X-JJ, L-JR, and SG revised the manuscript. J-PW and HH designed this work and agreed to be accountable for all aspects of the work.

## Conflict of Interest Statement

The authors declare that the research was conducted in the absence of any commercial or financial relationships that could be construed as a potential conflict of interest.
